# A Common Knowledge-Driven Generic Vision Inspection Framework for Adaptation to Multiple Scenarios, Tasks, and Objects

**DOI:** 10.3390/s24134120

**Published:** 2024-06-25

**Authors:** Delong Zhao, Feifei Kong, Nengbin Lv, Zhangmao Xu, Fuzhou Du

**Affiliations:** School of Mechanical Engineering and Automation, Beihang University, 37 College Road, Haidian District, Beijing 100191, China; zdlmgrzm9900@163.com (D.Z.); kong_fei@buaa.edu.cn (F.K.); lvnengbin@buaa.edu.cn (N.L.); xzmao720@buaa.edu.cn (Z.X.)

**Keywords:** machine vision, common knowledge, generic inspection framework, composite vision task, knowledge improvement

## Abstract

The industrial manufacturing model is undergoing a transformation from a product-centric model to a customer-centric one. Driven by customized requirements, the complexity of products and the requirements for quality have increased, which pose a challenge to the applicability of traditional machine vision technology. Extensive research demonstrates the effectiveness of AI-based learning and image processing on specific objects or tasks, but few publications focus on the composite task of the integrated product, the traceability and improvability of methods, as well as the extraction and communication of knowledge between different scenarios or tasks. To address this problem, this paper proposes a common, knowledge-driven, generic vision inspection framework, targeted for standardizing product inspection into a process of information decoupling and adaptive metrics. Task-related object perception is planned into a multi-granularity and multi-pattern progressive alignment based on industry knowledge and structured tasks. Inspection is abstracted as a reconfigurable process of multi-sub-pattern space combination mapping and difference metric under appropriate high-level strategies and experiences. Finally, strategies for knowledge improvement and accumulation based on historical data are presented. The experiment demonstrates the process of generating a detection pipeline for complex products and continuously improving it through failure tracing and knowledge improvement. Compared to the ($1.767^{{}^{\circ}}$, 69.802 mm) and 0.883 obtained by state-of-the-art deep learning methods, the generated pipeline achieves a pose estimation ranging from ($2.771^{{}^{\circ}}$, 153.584 mm) to ($1.034^{{}^{\circ}}$, 52.308 mm) and a detection rate ranging from 0.462 to 0.927. Through verification of other imaging methods and industrial tasks, we prove that the key to adaptability lies in the mining of inherent commonalities of knowledge, multi-dimensional accumulation, and reapplication.

## 1. Introduction

With the revival of computing power resources, connectionism, particularly in the form of deep convolutional neural networks (CNNs), has emerged as a powerful tool that provides automatic vision inspection (AVI) with stronger expressive power. This has greatly enriched the application of vision in automation and promoted intelligent manufacturing [1,2], especially in industries with batch and standardized production. The data-driven approach allows us to quickly construct models through annotations for specified tasks and achieve adaptation to the feature diversity. However, in the context of this prosperous development, the advancement of emerging intelligent perception and detection methods still faces great resistance in some high-end manufacturing industries with discrete attributes. Taking the assembly appearance quality inspection of complex integrated products in the small batch production mode as an example, the potential challenges can be summarized by the following three points:

1. Detectability of composite vision tasks under weak hardware constraints.

**Figure 1:**
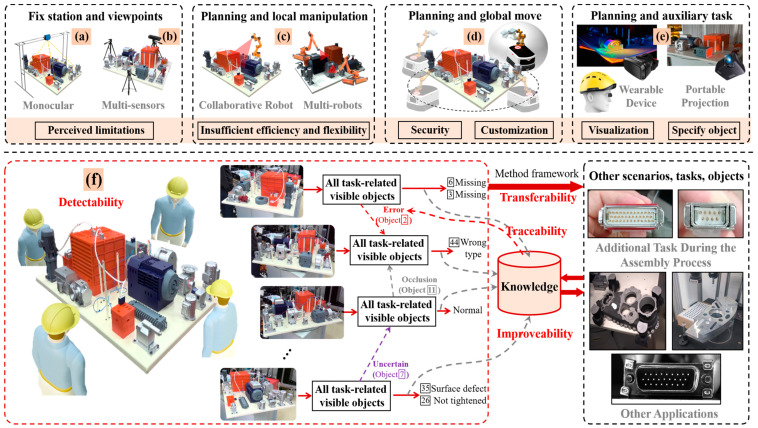
The challenges that current vision-based inspection will face in future manufacturing models are already emerging in the appearance quality assurance of the complex product assembly in high-end equipment manufacturing industry.

The customization and integration of products give them attributes, such as multiple objects, multiple states, extreme sizes, and varying features, that extend ordinary appearance detection into a composite vision task involving the adaptive extraction of task-related feature and differentiated demand guidance for pattern comparison. From the perspective of information capture, as shown in Figure 1f, constructing a collection method that allows us to capture data from multiple angles like humans is a prerequisite for ensuring object coverage. In terms of feature extraction, it is essential to overcome abnormal states, internal and external feature variations, and noise interference while achieving high-precision positioning of all elements (even though perspective effects can further amplify the size differences). In terms of pattern comparison, it is necessary to comprehensively analyze whether each object has anomalies based on the reference pattern under different degrees of occlusion from both qualitative and quantitative perspectives. If so, further identification of the type of anomaly is also needed. This demand for flexible capture makes many traditional industrial vision solutions [3,4,5] (as shown in Figure 1a–d) no longer applicable, and the demand for intelligent judgement makes some portable solutions (as shown in Figure 1e) only available for auxiliary visualization and active projection [6,7], while the analysis still relies on humans;

2. Traceability of internal failures and improvability of the inspection process.

To address diversity, we can train a scene-understanding model based on CNNs [8], even if the initial sample is small. However, the reliability of this data-driven approach is concerning. Specifically, the favorable performance of the model on prefabricated datasets may be masking numerous latent threats. It is important to ask whether the good performance on the test set reliably indicates that errors will not occur in the actual operation process, and determine what understandable adjustments can be made for possible prediction errors, to prevent repeating the same mistakes. Essentially, this touches on two important factors that are valued in high-end equipment manufacturing processes: traceability and improvability. There have been many excellent studies on the exploration of processing, production, scheduling, assembly, and workshops [9,10], etc., from the perspectives of digital twins and human-centered manufacturing. As an important aspect, however, research in AVI currently rarely discusses reliability issues, and rarely involves tracing the causes of generated errors, and improving inspection methods;

3. Low-cost transferability to other scenarios, tasks, and objects.

In addition to the appearance inspection of the product itself, the assembly stage also includes pre-assembly inspection of some components such as the appearance inspection of connectors. This additional work is a completely new task for the established method. Obviously, it is inconvenient to configure additional dedicated hardware imaging systems or specialized algorithms on the job site. In other words, assuming that we have devoted significant efforts and specialized skills to a detection task, it is important for the established method to possess the ability to allow people to get rid of the dilemma of starting from scratch when facing new applications. Furthermore, established methods and strategies should be transferred easily to provide knowledge for new scenarios and to facilitate re-adaptation [11,12].

To address these challenges, this article proposes a common, knowledge-driven, generic vision inspection framework, which allows us to generate an inspection pipeline suitable for composite vision tasks, accumulating knowledge and experience, and expanding to other scenarios and applications. The framework consists of three stages, namely, pattern connections based on industry common sense, the task-driven progressive perception of multi-objects, and knowledge-driven adaptive inspection. In addition, a knowledge improvement strategy is designed that allows for the interactive transformation of data into knowledge based on observation completeness, providing guidance on how to generate an inspection pipeline, improve inspection, and build a knowledge base with weak annotations for actual manufacturing process. The main contributions of this study can be summarized as follows.

1. We propose a generic inspection framework that includes pattern connection, progressive alignment, and adaptive detection to adapt to composite vision tasks and achieve universality and traceability;
2. We introduce embedding strategies that encompass knowledge of industry common sense, field-task knowledge, and expert experience to integrate learning models and knowledge models, ultimately enhancing adaptability and accuracy;
3. This study allows us to construct an inspection pipeline and accumulate data–knowledge–experience for different scenarios, tasks, and objects under weak annotation, and transfer to other applications;
4. Experimental results demonstrate that compared to end-to-end learning strategies, an inspection pipeline continuously optimized through fault tracking and knowledge improvements has higher performance potential and controllability.

The rest of this article is organized as follows. Section 2 reviews the significantly related works. Section 3 describes the proposed framework in three stages. Next, the experimental results and discussions are presented in Section 4. Finally, Section 5 concludes this study and presents future work ideas.

## 2. Literature Review

### 2.1. Generic Vision Inspection Framework

Tracing the progress of Industry 3.0 towards 4.0, the study of system engineering is essential for the informatization and intelligence of complex manufacturing tasks with knowledge-intensive characteristics [13,14]. In terms of AVI, ref. [15] proposed a framework based on 3D vision to solve the structural optimization of tunnels problem, and ref. [16] extended it through hierarchical planning and the structure from a motion pipeline. A component-aware abnormal detection framework was designed based on DL in [17], which can perform multiple adaptive and logical checks on some simple components. The AR-based projection was reconstructed by [18] into a three-stage structure for pose estimation, tracking, and correction. The software and its development framework for visual detection were designed in [19], which specifies the resources involved in AVI. In addition, DL-based detection pipelines for specific parts can be found in [20,21]. A general scheme was proposed by [22] for the appearance quality inspection of various types of electronic products with small size. However, these studies have not emphasized the role and commonality of knowledge, which limits the reusability of their frameworks in other scenarios or tasks.

### 2.2. Appearance Quality Assurance of Complex Product

The product appearance inspection generally refers to the detection of surface defects, which is a widely studied topic and can be regarded as a task of region sampling and local context pattern comparison [23]. However, the composite vision inspection task of complex assembled products complicates this process and transforms it into a cascading problem of high-accuracy pose alignment and multi-scale object pattern comparison.

In terms of alignment, the most advanced research emphasizing adaptability is represented in the field of pose estimation based on a learning paradigm with a 3D model. Research in this field can be divided into direct estimation [24,25,26,27,28,29] and indirect estimation [30,31,32,33,34]. According to the supervision strategy, it can also be divided into fully supervised [24,25,26,27,28,30,31,32,33,34], semi-supervised [35,36], and unsupervised [37,38] directions. In the unsupervised direction, some emerging topics focus on few-shot learning [39], multi-modal learning [40], and virtual–real domain adaptation [41,42], etc. Pose estimation is a difficult regression task and it is not easy to converge the training. The output of CNN is usually adopted as the initial solution, and a further exact solution can only be obtained by manual feature matching and optimization [29,43]. Other studies focus on offline viewpoint planning so that the alignment can be simplified through hardware and increase the practical feasibility [44,45]. However, in the actual implementation phase, its non-lightweight capacity and the deviations caused by motion errors and mechanism errors limit its popularization.

The pattern comparison described here essentially belongs to the cross-domain matching and difference metric of different information sources. The related research utilizes contrastive, matching, or metric learning strategies to perform comparisons in an encoded feature space [46,47], multi-level space [48], or source/target domain [49,50,51] and end-to-end. When the physical samples are sufficient, large models, such as high-performance target recognizers [52] and segmentation networks [53] can be directly migrated and applied through knowledge distillation [54]. Nevertheless, no matter the learning method, it is bound to face black box problems, annotation performance bottlenecks, and poor consistency of results, which has led to many applications in manufacturing still maintaining the knowledge-based manual models [55]. In addition, there is still a lack of research on standardized integration solutions for the two stages.

### 2.3. Knowledge Application for Vision-Based Methods

In recent years, it has gradually been discovered that the key to the successful application of this data-driven learning approach lies in the description of domain knowledge and the strategy of combining it with a learning paradigm. A review of relevant research indicates that the application of knowledge mainly includes the following aspects: (1) Datasets: simulation augmentation [56], empirically controlled sample construction [57], and automatic hard sample generation [58,59], etc.; (2) Network architectures: fusion learning of multi-modal data with complementary characteristics [60], contrastive, matching or metric learning with manually designed reference patterns [49,50,61], and designing special network layers to represent knowledge models [62,63], etc.; (3) Tasks: auxiliary tasks in multi-task learning [64,65], a priori based multi-task branch balance [66], etc.; and (4) Loss: physical model constraint terms [67], e.g., differential equations based on oscillation criteria [68], empirical model constraint terms [69], loss regularization terms [70], etc. In terms of knowledge representation, current research is primarily centered on the field of natural language processing (NLP) and its intersection with image understanding, focusing on solving knowledge graph modeling, recommendation systems, image text descriptions [71,72], etc.

Overall, existing knowledge applications are dedicated to describing specific objects or tasks and their combination with the components of DL module, while few studies focus on common knowledge in industry manufacturing and solve the integration of the experience paradigm and learning paradigm from the perspective of reliable system engineering.

### 2.4. Research Gaps and Motivation

Overall, visual technology can be abstracted as a process of processing perceptual information gradually and extracting task-related information through experience, rules, and external constraints. From this perspective, the core of adaptability to different scenarios, objects, and tasks lies in the extraction of knowledge commonalities, multidimensional expression, accumulation, and re-application. In a sense, this is not just a single technical issue. However, by reviewing existing work in the field of vision, it can be observed that there is a lack of mining common knowledge between scenes, as well as the lack of a generic framework that allows for the accumulation of knowledge and experience. The following observations can also be made: (1) In terms of unconstrained composite vision inspection for complex products, there is a lack of research on a cascaded overall framework for connection, alignment, and comparison. More emphasis is placed on point-based applications, such as pose estimation or defect recognition in local regions; (2) Inspection schemes need to be collaboratively improved in a controllable and reliable manner as data accumulates. However, the lack of a generic framework and standardized mechanisms makes it difficult to accumulate knowledge and experience. Therefore, methods are often limited to data preparation and model training, neglecting fault tracing and interactive improvement; and (3) The application mode of knowledge is still limited to the constraint design of specific networks and has not risen to a high-dimensional strategy to guide low-cost transfer between scenarios and tasks.

Motivated by these observations, this study proposes a generic, cascaded, visual perception and information processing framework driven by common sense, knowledge, and experience, which integrates learning modules and manual modules. Additionally, the application strategies, representation forms, improvement methods, and accumulation mechanisms of knowledge are explicitly summarized for different stages and methods. From the perspective of long-term practical significance, we hope that the proposed study and validation can stimulate more attention to framework research, multi-dimensional knowledge extraction, and knowledge improvement and accumulation.

## 3. Proposed Method

**Figure 2:**
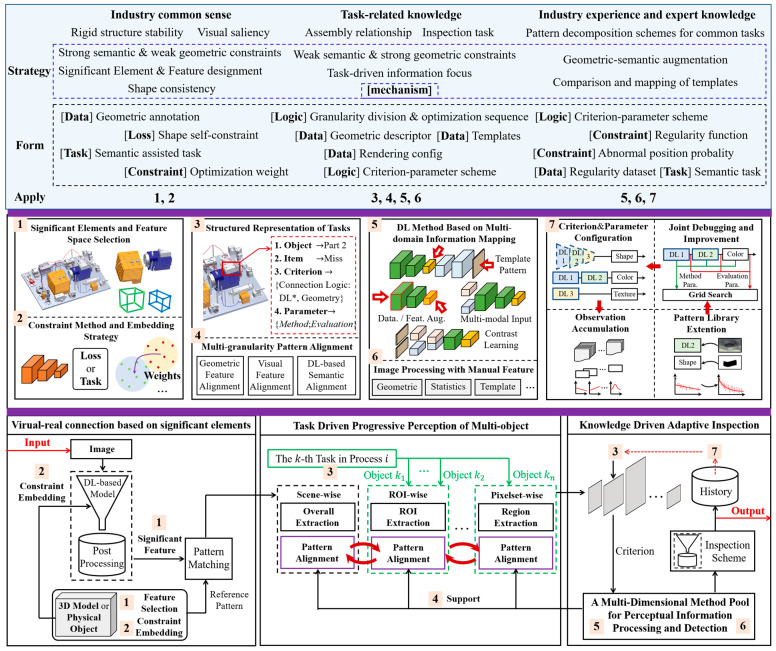
Overview of the proposed common knowledge-driven adaptive inspection framework. * abnormal.

The proposed framework is shown in Figure 2. The design of the framework execution mechanism is based on common strategies extracted from knowledge at different stages. Stage 1 is planned as a correspondence learning process anchored in a referential pattern, where common sense is seamlessly embedded through the provided form into the DL-based module via task-wise and geometric-wise constraints, etc., thus strengthening the learning purpose. Stage 2 is represented as a hierarchical pose refinement problem. To ensure accuracy, estimation is further extended to a multi-granularity object iterative matching and optimization model, assisted by multi-pattern alignment according to the task-related knowledge. Stage 3 is abstracted as a reconfigurable process of multi-sub-pattern space combination mapping and difference identification, through which expert knowledge and experience can be accumulated in the form of sub-pattern space combination strategies, processing parameter configurations and methods and data for failure simulation or augmentation. The numbers 1 to 7 represent the application of knowledge at different steps.

### 3.1. Virtual–Real Connection Based on Industry Common Sense

This stage aims to discover the commonalities between a given pattern (e.g., virtual model, template image, feature dictionary) and the physical space; establish a bridge for the exchange of non-homologous information; and facilitate the analysis of the physical object. To reduce the disturbance of multi-source noise on information processing and communication in this stage, it is crucial to identify the appropriate constraints and develop embedding strategies.

#### 3.1.1. Significant Elements and Feature Space

Significant elements S refer to objects that have advantages in size, visual information, and contextual distinguishability with the given scene such as boundaries in PCBs [73], holes of aircraft skins [74], markers on scenarios [75], and designated contours in parts [76]. Determining the feature space means that significant elements are expected to be transformed into a domain independent public space to build correspondence with a given pattern by, for example, encoding elements into 2D, 3D [77], or pose [43] geometric space to perform matching with a given pattern (e.g., pointset, 3D model) [55]. Alternatively, semantic features of elements can be extracted and mapped to the target pattern space [49] or a common space [78] to complete recognition, alignment, positioning, etc.

#### 3.1.2. Constraints Method and Embedding Strategy

The core of this step lies in the mining of rules with inherent invariance; we recommend rigid body structure invariance in industrial manufacturing. For the DL part, constraints are applied to the loss in the form of tasks or regular terms, while for the non-DL part, constraints are employed to adjust the execution process in the form of empirical parameters.

**Figure 3:**
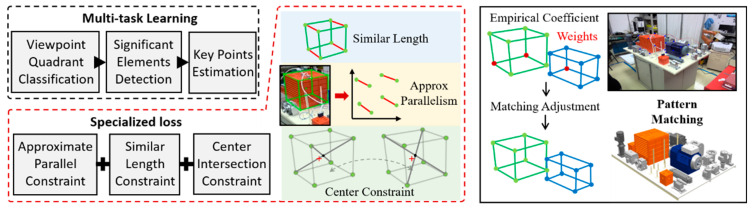
Example of constraint-embedding strategy for pattern connection.

For the sake of illustration, as shown in Figure 3, we designed a significant 3D box estimation model based on [30] that integrates shape self-constraint. In the multitasking mode, according to the concept of focusing attention, quadrant classification, target recognition, and point estimation can be selected for constraint construction to aggregate information. The loss L can be formulated as $L=w_{m}L_{m}+w_{c}L_{c}+w_{r}L_{r}$, where $w_{*}$ is the branch balance coefficient, $\left\{L_{c},L_{r}\right\}$ can be derived based on common classification and recognition tasks [46] and as follows:

(1) $$L_{m}=\sum_{i\in S}\left\{\sum_{j\leq 9}\left\|f^{t}\left(I,\Theta \right)-{gt}^{t}\right\|+\sum_{m}\left(\left(1-f_{m\in {gt}_{cell}}^{c}\right)+f_{m\notin {gt}_{cell}}^{c}\right)\right\}.$$

Point regression loss and cell-oriented confidence loss are adopted in (1), where $s=f^{t}\left(I,\Theta \right)$ represents the coordinate obtained through neural network f with parameter Θ, gt denotes coordinate annotations, and cell depends on the size of the output. In terms of regular terms, $L_{m}$ can be further extended as follows:

(2) $$L_{m}^{\prime }=L_{m}+{\{\lambda }_{P}L_{P}+{\lambda_{L}L}_{L}+\lambda_{C}L_{C}\},\;\;$$

where $\lambda_{*}$ is a set of trade-off coefficients. $L_{P}$ considers the feature of close size, and can be formulated as follows:

(3) $$L_{P}=\sum_{S}\frac{1}{\left|set\right|}\sum_{G\in \left\{set\right\}}\sum_{\begin{array}{c} g_{i},g_{j}\in prio \end{array}}max\left(arcos(g_{i},g_{j})-\tau_{P},0\right),\;$$

where g∈G denotes the manually specified segment between two s∈s, set describes the set of selected segments, prio represents a prior knowledge that depends on the shape of the product. $L_{L}$ emphasizes the approximate parallelism of specific structures and can be described as follows:

(4) $$L_{L}=\sum_{S}\frac{1}{\left|set\right|}\sum_{G\in \left\{set\right\}}\max\left(\frac{1}{{\bar{g}}_{G}}\sqrt{\sum_{g\in G}{\left(\left|g\right|-{\bar{l}}_{G}\right)}^{2}/(\left|G\right|-1)}-\tau_{L},0\right),{\bar{g}}_{G}=\frac{\sum_{g\in G}\left|g\right|}{\left|G\right|},$$

$L_{C}$ explores the relationship between the centroid of a rigid body and its shape as follows:

(5) $$L_{C}=\sum_{S}\frac{1}{\left|set\right|}\sum_{G\in \left\{set\right\}}\sum_{\begin{array}{c} g\in prio \end{array}}max\left(d(c,g)-\tau_{C},0\right),$$

where $d\left(c,g\right)$ measures the distance between the center c and a segment g, $\left\{\tau_{P},\tau_{L},\tau_{C}\right\}>0$ are used to characterize tolerance degree. In addition, these knowledges can also be modified according to actual situations, such as [79]. Once the physical samples are insufficient, domain adaptation [41] or dataset augmentation [58] can be supplemented. Additionally, when it is difficult to simplify the representation of geometric invariance, operator can refer to an end-to-end learning approach [25] or implicitly embed constraints in the form of multi-modal learning [37].

In the non-DL part, post-processing includes, but is not limited to, outlier filtering, and the correction of missed detections [80]. Then, the refined significant feature $s^{\prime }$ is fed into pattern matching, and this work can be empirically guided by weight coefficients. For example, the conf of s estimated by DL and the visibility of s can be combined to initialize weight comprehensively to assist in virtual and real pose alignment as follows:

(6) $$\left(R,t\right)=\underset{R,t}{\mathrm{argmin}}\left\{\sum_{i}g_{s_{i}}\left(v,conf\right)\left\|T_{R,t}\left(s_{i}\right)-p_{i}\right\|\right\},\;$$

where $g_{s_{i}}\left(\cdot \right)$ is an empirical function, $p_{i}\in p$ represents the i-th item in the reference. In 2D matching,$R\in R^{2\times 2},t\in R^{2\times 1}$, $T_{R,t}\left(s_{i}\right)=Rs_{i}+t$, and (6) can be solved by [81]. For pose estimation, $R\in R^{3\times 3},t\in R^{3\times 1}$, $T_{R,t}\left(s_{i}\right)=\pi \left\{K\left(Rs_{i}+t\right)\right\}$, (6) can be solved using [82], where K refers to the camera matrix and π denotes the de-homogenization operation.

### 3.2. Task-Driven Progressive Perception of Multi-Object

This section aims to construct a task driven complex assembly product pose adjustment model to achieve multi-scale object localization and accuracy adaptation under background and local abnormal disturbances.

#### 3.2.1. Structured Representation of Task

**Figure 4:**
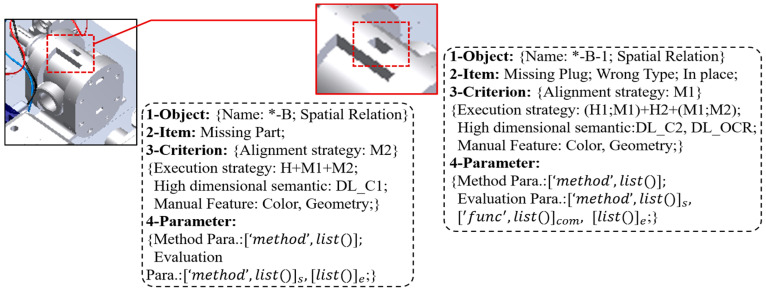
Structured task representation model.

As shown in Figure 4, we design a structured task representation model based on a quadtree mode that endows the inspection framework with task-driven mechanism. **Object** provides “**name**” and “**spatial relation**”. “**name**” can be used to create learning labels and build association in data structured management. “**spatial relation**” describes the relationship between object and scene, which can be expressed as coordinate system transformation, topological relationship, etc. The expected detection task is arranged in **Item**. **Criterion** defines the alignment and detection scheme for **Item**. For example, assuming a method pool containing multiple DL models and image processing algorithms has been constructed. Then for “*-B”, a classification DL model, a color algorithm, and a geometric algorithm are manually configured. The “**+**” in the “**Execution strategy**” indicates the execution order of the three. Next, for each method, “**Method Para**”. in **Parameter** defines the internal parameters of the execution, “**Evaluation Para**”. is the validity parameter adopted to evaluate whether there is a need to continue between “H” and “M” or “M” and “M”, and ${\left[.\right]}_{e}$ is employed to determine whether there are omissions.

#### 3.2.2. Multi-Granularity Pattern Alignment

**Figure 5:**
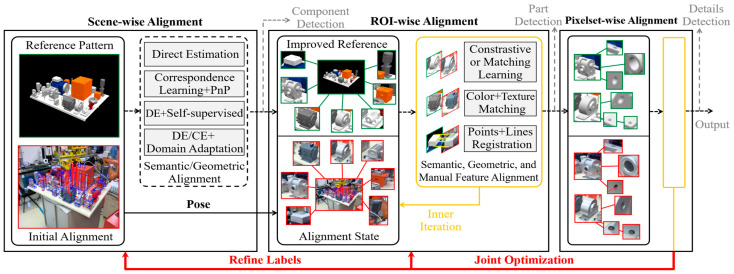
Multi-granularity Pattern Alignment Pipeline.

Accurate extraction is essentially a high-accuracy geometric estimation problem. The estimation can be reconstructed as an alignment problem under given reference pattern constraints. Furthermore, as the required accuracy increases, the reference pattern also needs to be improved (re-rendering image, expanding template library), at which the focus of alignment shifts to the formulation of multi-spatial invariance mining and collaborative optimization. To this end, taking the connected virtual-real as the initial state, a multi-granularity pattern alignment pipeline for positioning of multi-scale objects is designed as shown in Figure 5 (3D, 2D can be regarded as a simplification of this problem).

Assuming that the objects involved in the appearance inspection of a product include large-sized components (LSC), medium-sized parts (MSP), small-sized accessories (SSA), and specific details (SD) (e.g., holes, faces). The perception process can be summarized based on task characteristics as follows:

**1. Mainly LSC, with a few MSP (e.g., robotic assembly, robot grasping, AR/VR)** Low accuracy requirement ($>5^{{}^{\circ}},10cm$) means that more effort can be allocated to cope with feature variation, and the corresponding data-driven learning strategies are listed in Figure 5. If the geometric feature is difficult to explicitly define, an end-to-end direct estimation [24,26,27,28] can be adopted to infer pose or relative pose as follows:

(7) $$L=\sum_{t\in T_{a}}\left\{f_{T_{a}}^{a}\left(\;f_{\begin{array}{c} x\in X_{T_{a}}\\ \Theta \in \Omega_{T_{a}} \end{array}}^{e}\left(x,\Theta \right),\ldots \right)-{gt}_{T_{a}}\right\}+\left\{f_{T_{m}}^{a}\left(\;f_{\begin{array}{c} x\in X_{T_{m}}\\ \Theta \in \Omega_{T_{m}} \end{array}}^{e}\left(x,\Theta \right),\ldots \right)-T({gt}_{T_{m}})\right\}\;\;$$

where $T_{m},X_{T_{m}}$ denote the main task and input set (e.g., image, depth), $T_{a},X_{T_{a}}$ consist of auxiliary tasks (e.g., depth prediction, target recognition, foreground segmentation [30], etc.) and their inputs,$f^{e},f^{a}$ are feature encoder and aggregator, respectively, and T is a converter used to re-parameterize pose for CNN (e.g., quaternion, [29], etc.). On the contrary, the estimation can also be indirectly achieved by learning sparse or dense geometric correspondence, in which ${T(gt}_{T_{m}})$ in (7) can be replaced by offset [25], vector distribution [31], 3D coordinates [32,33], intermediate representation [34], etc. On the other hand, considering the difficulty of preparing high-quality dataset, research combining self-supervision and domain adaptation has gradually drawn more attention.

The self-supervision promotes alignment learning by manually designing domain independent consistency evaluation rules [37], which is summarized in “4” of Figure 2 and can be described as $L_{self}=L_{geo}+L_{feat}+L_{sem}$,

(8) $$L_{geo}=D_{geo_{2D}}\left(G_{2D},{rf}_{2D}\right)+D_{depth}{\left(G_{depth},{rf}_{depth}\right)}_{v}+CD\left(G_{3D},{rf}_{3D}\right),\;\;$$

(9) $$L_{feat}=D_{{feat}_{co}}\left(F_{co},{rf}_{co}\right)+D_{{feat}_{tex}}\left(F_{tex},{rf}_{tex}\right),co\in Rgb,Lab,\ldots ,\;\;\;$$

(10) $$L_{sem}=\sum_{layer}\{D_{sem}({ft}_{layer},{rf}_{layer})\}.$$

In different representation spaces, (G,F,ft) are the generated results of the input pattern, and ${rf}_{*}$ belongs to the reference pattern. Thus (8) includes 2D rules (e.g., contour, mask), depth rule, and 3D rules, where $D_{depth}{\left(.\right)}_{v}$ indicates that only the visualized body can be considered, and CD is used to measure point cloud differences (e.g., chamfer distance). The similarity of the LAB space [83], structural similarity in the RGB space [84], and texture consistency are modeled in (9). As expressed in (10), it is also reasonable that the semantics of different patterns in the corresponding network layers should be similar. The domain adaptation performs the distribution alignment of a given pattern to the target pattern in the semantic space through technologies such as GAN [38]. This research can be seen as an extension of the former, targeting to replace manual embedding rules through end-to-end learning.

**2. Mainly LSC, MSP, with a few SSA, SD (e.g., Electronic/Electromechanical Products)** In some large scenarios, the results of DL adjustment in a) may still not satisfy the requirements, which means that improving alignment accuracy requires more fine-grained element support and synchronous improvement of the current reference. Therefore, in the second stage of Figure 5, three basic alignment strategies are provided. When an object appears frequently but its features are not prominent, CNN-based learning paradigm can be maintained (e.g., metric learning [85], matching learning [86]) to identify the differences between the extracted region of interested (ROI) and its reference (e.g., virtual ROI). Otherwise, for ROIs with low information, manual image features and geometric features will be better choices.

Define a complete alignment process between input ROI $x_{k}$ with its reference pattern ${rf}_{k}$ as follows:

(11) $$ref≜w_{C}f^{C}\left(x_{k},{rf}_{k},\Theta_{C}\right)⊕\left(1-w_{C}\right)f^{M}\left(x_{k},{rf}_{k},\Theta_{M}\right),\;\;$$

$$\left(R^{*},t^{*},s^{*}\right)={argmin}_{R,t,s}\left\{\sum_{i}\left[D_{T}\left(T_{\Omega }\left(p_{i}\right),T_{\Omega }\left(t_{i}\right)\right)\right]\right\},$$

(12) $$p_{i}\in G_{k,2D},t_{i}\in {rf}_{k,2D}^{\prime },\;{rf}_{k,2D}^{\prime }={rf}_{k,2D}⊕ref,\;R\in R^{2\times 2},t\in R^{2\times 1}.$$

ref defines a state correction strategy, where $w_{C}$ is the attention and $f^{C},f^{M},\Theta_{C},\Theta_{M}$ represents the methods and their internal parameters. For example, the region most similar to ${rf}_{k}$ within $x_{k}$ can be extracted by matching CNN $f^{C}$ and the matching vector can be obtained. Similarly, $f^{M}$ is the template matching. In this way, ref is the weighting of two potential directions, by which we can adjust the geometric feature ${rf}_{k,2D}$ of ${rf}_{k}$ to form ${rf}_{k,2D}^{\prime }$. Equation (12) describes a generalized model for point set registration, where $\left(R^{*},t^{*},s^{*}\right)$ is a set of 2D transformation parameters, $T_{\Omega }$ represents a mapping approach with implicit parameter Ω (e.g., weight mapping [81], kernel function mapping [87], mixed Gaussian mapping [88], etc.). $D_{T}$ refers to the measurement established on T (e.g., Euclidean distance, kernel correlation, probability likelihood, etc.).

In such way, define any nearest neighbor point of $t_{i}^{*}\in {rf}_{k,2D}^{*},\;{rf}_{k,2D}^{*}=R^{*}(s^{*}({rf}_{k,2D}^{\prime }-{\bar{rf}}_{k,2D}^{\prime })+{\bar{rf}}_{k,2D}^{\prime })+t^{*}$ as $p_{i}$. $\left\{P_{k},P_{k,3D}\right\}$ is the set of all $p_{i}$, and its 3D coordinate. Then, in the ROI-wise internal loop, the input pose and reference pattern can be updated through the ${rf}_{k,2D}^{*},P_{k,3D}$ of all ROIs. In the external loop, this stage can be used to synergistically adjust the labels of the previous stage.

**3. LSC, MSP, SSA, and SD are all required. (e.g., this case or a similar product).** This type of task means that any interested details may not be allowed to deviate from the alignment process, even if the current performance has significantly exceeded the common indicators $\left(5^{{}^{\circ}},5cm\right)$ adopted in the CV field. In other words, the internal loop Optimization in b) needs to be performed again on the details associated with inspection items and manually selected auxiliary features (e.g., holes, vertices). It should be noted that we recommend $ref≜f^{M}\left(x_{k,j},{rf}_{k,j},\Theta_{M}\right)$ as DL is not suitable for small objects. Equation (12) can be simplified as an isolated point-to-point relationship $\left({rf}_{k,j,2D}^{*},p_{k,j},p_{k,j,3D}\right)$, where j is the j-th detail of ROI $x_{k}$. Then, each ROI will be bound to its details, further achieving joint optimization.

**Figure 6:**
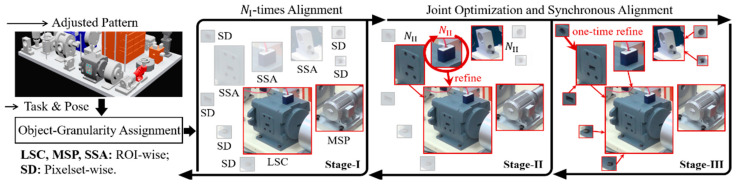
A complete joint optimization of objects with different granularities.

A complete process is presented below and explained in Figure 6:

(13) $$I≜{op}_{ali}^{N_{I}}\left\{{rf}_{k}^{*},P_{k}|k\in \left\{LSC\cup MSP\right\}\right\},\;\;$$

(14) $$II≜{op}_{ali}^{1}\{({rf}_{k_{1}}^{*},P_{k_{1}});{\left({rf}^{*},P\right)}_{k_{2}}|k_{1},k_{2}\in \{LSC\cup MSP\}\}\;,$$

(15) $${\left({rf}^{*},P\right)}_{k_{2}}=\{({rf}_{k_{2}}^{*}⊕{ref}_{m},{rf}_{m}^{\prime }⊕{ref}_{m},P)|m\in {SSA}_{k_{2}}\},$$

(16) $${ref}_{m}={op}_{2D}^{N_{II}}\left\{\left(G_{m},{rf}_{m}\right)|m\in {SSA}_{k_{2}},I\right\}.$$

${op}_{ali}^{1}$ represents performing a pose optimization, updating reference pattern, and aligning features. ${op}_{2D}^{N_{II}}\equiv$ Formula (12). Numeral III is similar to II, except that ref is directly determined by single-point matching. The above work establishes an integrated association of task–object–perception, and ensures the accuracy and reliability of extraction at different granularities.

### 3.3. Knowledge-Driven Adaptive Inspection

**Figure 7:**
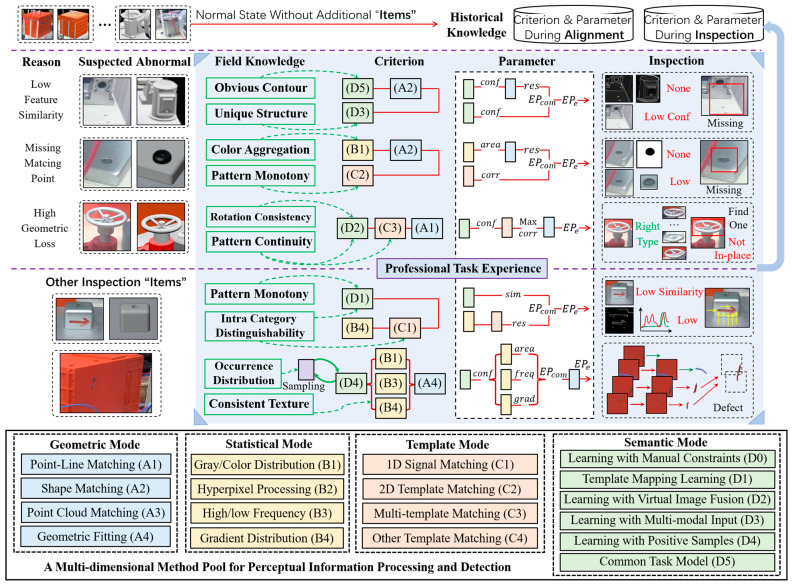
A Knowledge-based adaptive inspection scheme for perceived multi-size objects.

Theoretically, all the components to be detected participate in alignment, which means that their processing data will provide the basis for inspection. Some potential anomalies that were overlooked during alignment due to inappropriate results will be further confirmed in this section. Therefore, as shown in Figure 7, this section introduces a knowledge-based adaptive inspection scheme for perceptual objects.

#### 3.3.1. Method Pool

A multi-dimensional method pool is constructed based on industrial vision knowledge (e.g., objects, features, and possible methods), which includes geometry, statistics, template, and semantics. In each mode, we provide general strategies for information with different dimensions. Due to the mature development of manual feature processing, only DL-based strategies listed in the semantic mode are emphasized here.

“**D1**” assumes a monotonic reference pattern and constructs data-mapping learning to establish a matching bridge between the perception and the target information. According to the type of mapping space, it can be further divided into an encoding space [89] and reconstructed space [49] as shown in “5” in Figure 2. Fusion-based perceptual enhancement has proven to be an effective strategy for improving CNN performance. There are many sources of manufacturing information that can be integrated, among which the most commonly used are images (“**D2**”) and point clouds (“**D3**”).

According to different fusion strategies, it can be divided into data-wise fusion (e.g., mixed virtual real dataset), and feature space fusion [78,90]. In practice, it is particularly important to note that the fused information should be able to improve intra-class consistency and inter-class distinguishability.

For rare or unseen anomalies, “**D4**” suggests a way to learn from positive samples [91], with a focus on whether the learned prototype under imbalanced categories is close enough to the true distribution. For industrial inspections that emphasize reliability, it is recommended to strictly control the information received by “**D4**” (e.g., accurate positioning) to prevent unexpected shifts in distribution caused by interference. In addition, CNN models for general tasks also have high application value, such as [92]. These approaches encode perception into an information space that humans can understand, enabling many traditional algorithms to cope with complex contexts.

#### 3.3.2. Inspection under Different Situations

In theory, as long as the object has not been omitted or obvious incorrectly installed, its matching results will exhibit high consistency in multiple stages during alignment. Otherwise, situations, such as low similarity or no matching point, may indicate anomalies. To verify these suspected anomalies, as shown in the second line of Figure 7, we summarize the following representative cases based on field knowledge from aspects such as information volume, internal pattern, contextual consistency, regularity, etc.:

**1. Components with surface treatment.** The internal texture of surface-treated components is difficult to recognize, and distinction from the context can be achieved through contour feature or external information. “**D5**” will be a good strategy to provide robust edges when the contour is difficult to manually extract, otherwise conventional operators like Canny can be directly applied [55]. When the context is difficult to distinguish, “**D3**” enhances its discriminative ability by incorporating 3D information, enabling end-to-end estimation of the target state;

**2. Fasteners/Accessories.** As small objects, fastener-like targets typically exhibit high texture clustering and limited feature variation. These attributes permit the examination from two distinct aspects: color and shape, as well as pattern similarity;

**3. Parts with rotational similarity.** To ensure inspection reliability, a robust **Criterion** is provided here. Virtual–real metric learning in “**D2**” can generally provide coarse-grained presence recognition. If the local pattern changes of an object exhibit continuity and regularity across different global viewpoints, a strategy of reference pattern sampling and multi-space fusion can be adopted. Specifically, semantics from different viewpoints can be fused in the semantic space, or multiple templates can be predesigned in the feature space. In addition, to address the contradiction between the estimated state and the abnormality in alignment, a more targeted geometric strategy “**A1**” is assembled to identify any improper installation, as shown in Figure 7;

**4. Defects on small-sized objects.** Precisely extracted small objects or regions have limited information content, and any abnormality will cause significant pattern differences. In such cases, an acceptable defect recognition result can be obtained by template-based mapping learning with few-shot learning [49]. In addition, multi-directional gradient information extraction and signal matching can also provide a reliable basis for defect localization;

**5. Defects on large surfaces.** Defects that occur on large flat surfaces on a component or a specific area of an assembled product are generally unpredictable in location and appearance. To prevent small defects from being indistinguishable, joint sampling and comparison is a widely adopted approach [93]. In this study, the occurrence distribution obtained from production experience can be used to guide sampling, and the texture consistency model can assist in judgment from aspects such as gradient, frequency domain [94], etc. The initially adopted CNN can be learned through “**D4**”. With the accumulation of historical data in the framework, the CNN can gradually be adjusted to a classification model targeting negative samples;

**6. Foreign object debris.** There is limited research on this topic, mainly due to the challenges in vision-based locating of foreign object debris in a complex assembled product, and its recognition falls under the category of open-set problems [3]. Nevertheless, this task can be transformed into a joint task of empirical sampling and pattern comparison within this framework. For comparison, we suggest choosing “**D5**+**A1**”, “**D2**+**A3**”, “**D3**” or a combination of them.

#### 3.3.3. Knowledge Improvement Strategies

The framework presented in this paper can be regarded as an intermediary operating within the current manufacturing phase and knowledge space. The processing results of various stages on perception are continuously accumulated in historical data. Essentially, these results reveal the evolutionary regularity of information in the pattern spaces associated with various methods. To improve the observation of the regularities generated in each pattern space, it is crucial to collect alignment and detection data during the trial operation phase.

**Figure 8:**
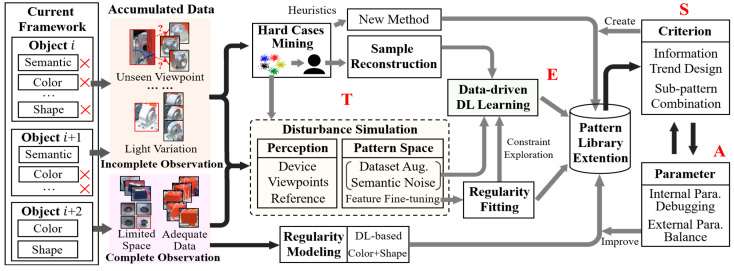
TESA knowledge improvement strategy. The marker × is used to indicate whether the observations in the corresponding feature space are complete.

Here, as shown in Figure 8, we demonstrate an interactive hybrid iteration strategy that follows the principles of technological improvement, experience accumulation, strategy adjustment, and adaptation (**TESA**), converting accumulated data into knowledge for inspection. Specifically, the data generated by the current framework can be divided into two parts based on performance and the descriptive ability of each sub-pattern space, namely, complete observation, and incomplete observation:

1. **Complete observation.** This means that the current observation can be well-represented by the configured sub-pattern (or combination), while the subsequent addition of data has little impact. For example, the presence or absence of fasteners can be determined using typical templates or area-based conditions. Due to good visibility and fewer unpredictable changes, some objects can be modeled by CNN and existing datasets to improve their sub-pattern (semantic);
2. **Incomplete observation.** Essentially, incomplete observation is a challenge that less constrained vision-based applications will inevitably face. In other words, any changes in Man–Machine–Material–Method–Environment (4M1E) may lead to deviations in perception that are difficult to be covered by the pattern space constructed by existing knowledge such as an unseen viewpoint. In this situation, if the difference with the internal sub-pattern space is too large, then manual intervention is necessary. For the semantic space, difficult case mining and augmentation [57,59] has been proven to be an effective strategy. For other spaces, exploring new sub-patterns or reconstructing existing patterns can be considered;
3. **Intermediate state.** More objects belong to the intermediate state between (1) and (2). For these data, a combined disturbance strategy oriented towards perception and pattern space is adopted here to simulate potential variation based on existing knowledge, as follows: (a) **Perception**. Perform pose perturbation around the viewpoint of the existing sample and simulate possible geometric matching differences using virtual rendering. For example, in the small part recognition, geometric dictionaries under different states can be constructed to improve the perception by perturbing the local virtual viewpoint; For foreign object debris, normal patch collection can be carried out around the frequently occurring areas on the data after viewpoint perturbation, to improve observation of reference pattern and enhance metric ability; and (b) **Pattern**. For high-dimensional semantic features, it is recommended to use data augmentation strategies and feature layer Gaussian noise, while for low-dimensional manual features and modeling functions, it is recommended to use internal parameter perturbations. For example, in the fine-defect detection task of small objects, recognition results obtained through manual rules or empirical functions based on multiple factors, such as shape, texture, and geometric attributes, may fail due to insufficient consideration of abnormal changes in these factors. Therefore, establishing disturbance and simulation (e.g., shape deformation, feature space noise) based on intermediate results at each stage can enhance the adaptability of the method to new patterns. Then, re-train or refit (cluster) the data after perturbation and embed the discovered inherent invariance as constraints into the learning process.

Given the updated pattern library (including fine-tuned DL models, re-fitted functions, expanded feature libraries or dictionaries, supplementary template images, and adjusted parameter sets), **Criterion** and **Parameter** are then locally adjusted object by object as shown in number 7 of Figure 2. Subsequently, the improved **Criterion** and **Parameter**, are integrated, as well as the expanded pattern library, into the entire framework for joint refining with **Connection** and **Alignment**. For example, in the new alignment process, we can incorporate small-sized objects that were not previously considered to enhance alignment accuracy.

## 4. Case Study

### 4.1. Preliminary Work

#### 4.1.1. Scenarios and Datasets

**Figure 9:**
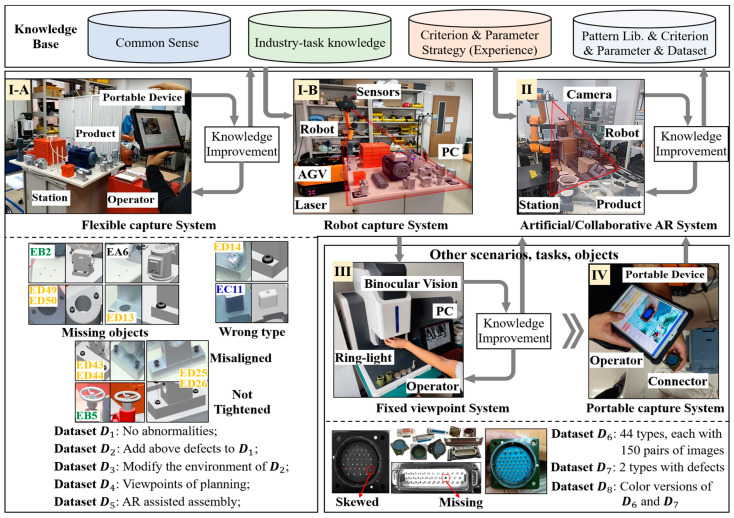
System configuration and related datasets for each scenario.

**Overall idea:** As shown in Figure 9, to simulate the real-world problems faced in the actual manufacturing process, we will start from an experimental platform containing a small amount of available data. By continuously introducing new samples, tracing failures, and improvement to enhance performance and accumulate knowledge. During this period, we will gradually demonstrate its performance comparison with state-of-the-art deep learning methods under ideal conditions. Furthermore, based on existing knowledge, low-cost migration to other imaging approaches, scenarios, and objects can be achieved through **Criterion** planning and **Parameter** fine-tuning.

**Figure 10:**
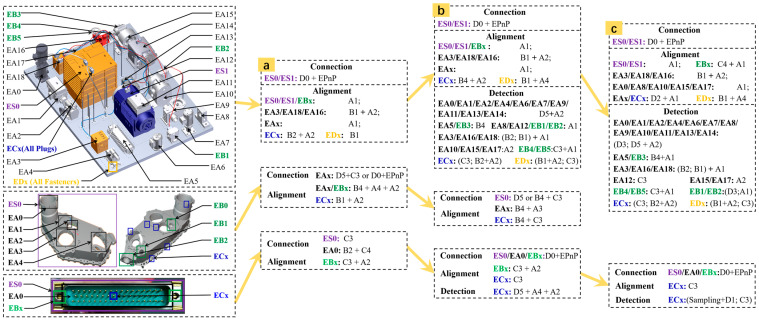
Object ID and **Criterion** evolution: (**a**) connection, alignment, and data accumulation; (**b**) initialization of inspection system combined with DL module; and (**c**) a complete solution that emphasizes reliability.

**Scenarios:** The construction of scenarios, tasks, imaging, datasets, etc., is shown in Figure 9. **Scenario I:** a complex assembled product with 113 components was constructed, where the objects were further divided by size (ES~ED) as shown in Figure 10. The product was placed in a complex background, and its appearance is inconsistent from different viewpoints. Some equipment and parts were surface treated to exhibit low texture and high color consistency. To verify universality, two common imaging pathways (I-A, I-B) were arranged here; **Scenario II:** Robot with fixed station was deployed to perform multi-view imaging of a structural component, targeting to identify the positions of these holes; **Scenario III:** 46 kinds of electrical connectors were prepared, which include 6 types of standard parts (pins), more than 40 different arrangements and 4 installation approaches. Besides, the experimental platform simply adopted the binocular vision, and was equipped with low-angle ring lights, without any special design or servo control system; and **Scenario IV:** Products of **Scenario III** were captured by an industrial tablet.

**Inspection Task:** The task is to identify the assembly status of all objects in **Scenarios I**-**A, -B,** and check for any omissions, wrong types, or incorrect positions (misalignment, not tightened, or skewed). For **I-A**, this task involves overcoming disturbances posed by the background, lighting conditions, and self-occlusion while addressing high-accuracy positioning based on monocular image and virtual-real comparison of multi-scale objects. In **II**, rapid and minimally costly deployment and accuracy assurance are the focus of this common AR task. For **III** and **IV**, it is necessary to demonstrate adaptability to variations in appearance and imaging posture across different models, and to achieve accurate identification of products and their densely packed small targets.

**Dataset:** In **I-A**, $D_{1}\sim D_{3}$ were built by capturing a handheld portable device around the platform at different pitch angles. According to the artificial markers, the ground truth of the pose and ROIs were calculated. $D_{1}$ contains 89 images with no abnormalities, while $D_{2}$ contains 112 images, which are configured with defects for specific EA-ED objects. After modifying the illumination and background, 80 more images were captured as $D_{3}$. The number of visible defects is manually counted and listed in Table 1.

$D_{4}$ contains 20 images captured by mobile robot; $D_{5}$ contains 60 images captured by collaborative robots. The configuration of $D_{6}$ is shown in Figure 9, and two types with artificially set missing and skewed pins are arranged in $D_{7}$. The color image of each model connector was prepared in $D_{8}$.

**Indicators**: Euler angle error ${\bar{e}}_{R}^{*}$, translation error ${\bar{e}}_{t}^{*}$, positioning performance ${\bar{IoU}}^{*}$, normal state ${\bar{r}}_{n}^{*}$, missing part ${\bar{r}}_{m}^{*}$, wrong type ${\bar{r}}_{w}^{*}$, incorrect location ${\bar{r}}_{i}^{*}$, where ∗ can be considered as D or specific object, etc.

#### 4.1.2. Criterion and Method Pool

Figure 10 depicts the configurations of **Criterion** for different scenarios, which are based on the knowledge of the intersection between vision and manufacturing summarized in Section 3.3.2.

It should be noted that, only the inspection logic was presented here, based on which different specified methods would be arranged during the experimental phased to demonstrate the universality and scalability of the proposed framework. Additionally, the initial **Parameter** is set based on the actual processing results of each stage, and not be elaborated here. Then, the **Criterion**, **Parameter**, and the above tasks will be encapsulated as quads and associated in XML with the given process 3D model.

### 4.2. Evaluation on Complex Assembled Product

#### 4.2.1. Deployment Work

In this section, for **I-A**, datasets $D_{1}\sim D_{3}$ were, respectively, used for simulating prototype system construction (PSC), trial operation (TO), and practical application (PT). Unlike the prevalent practice in the computer vision field that relies on extensive pre-prepared datasets, this study initially assumed that only images of PSC and labels (may contain errors caused by occlusion and intuitive annotation) of ES0 and ES1 could be used. The framework construction detail is listed in Table 2. The network used in D2 refers to [61], where the task branch is modified to object recognition to assist in identifying abnormal states. D3 adopts depth-data processing branch and performs feature fusion according to [89], maintaining consistency with D2 in the task. In D5, a lightweight contour prediction network is constructed based on [95].

Based on the TESA concept, the experiment is divided into four stages as shown in Table 3. Each stage is required to be tested on PT to demonstrate the current potential.

#### 4.2.2. Validation under Possible Non-Ideal Conditions in Actual Production

**Stage-A.** Five representative pose estimation methods were used as control groups. Specifically, **Group I** learned indirect relationships through [30] and EPnP, **Group II** and **III**, respectively, improved the results of **Connection** and **Group I** using poseNet2 [29] and pose ground truth. **Group IV** employed the advanced estimator PVNet [31], in which semantic labels were generated from the minimum bounding rectangle of significant geometric feature. **Group V** arranged the unsupervised learning method self6D [37], where the depth map is replaced by the simulation data with pose ground truth, and the differentiable renderer was pytorch3D [100].

**Figure 11:**
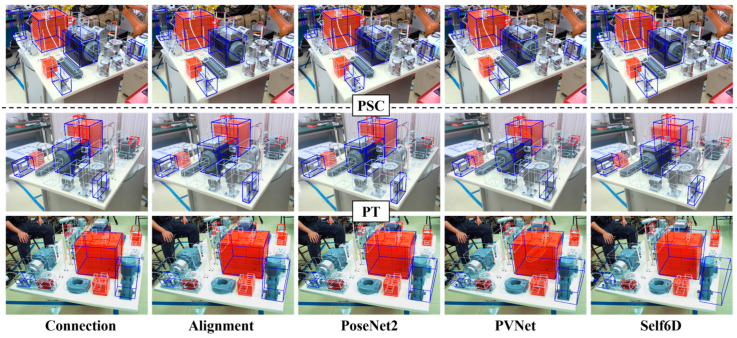
Examples of the estimation performance of different groups. The red/blue boxes are used to visually display accuracy.

The performance of each group on different datasets is listed in Table 4, and some examples can be found in Figure 11. The comparison of the performance between **Connection** and **Group I** indicate that embedding prior knowledge or industry-specific rules into CNN in the form of constraints can yield significant benefits. The results of **Group II** and **III** demonstrate the fine-tuning ability of supervised mode. However, although the accuracy achieved is enough for grasping, tracking, or AR, it is still slightly insufficient for a complex inspection task. **Group IV** is not constrained by the limitations of manual geometric representation, thereby achieving better results. Nevertheless, the unsatisfactory alignment performance in the surrounding region shown in Figure 11 shows that accurate estimation based solely on a few elements is not sufficient for such a complex case. Although the performance of unsupervised learning is inadequate, the constraint strategy emphasized in self6D highlights the potential of this approach in industrial manufacturing. In PSC, for applications where actual datasets are difficult to obtain, the **Connection** or the first phase of **Alignment** in this framework can be modified to an unsupervised form with knowledge constraints. On the other hand, the performance changes on PSC and PT demonstrate the stronger adaptability of learning paradigms to pattern changes, which also implies the necessity of the transition from knowledge-based agent to learning agent emphasized in **TESA**.

**Figure 12:**
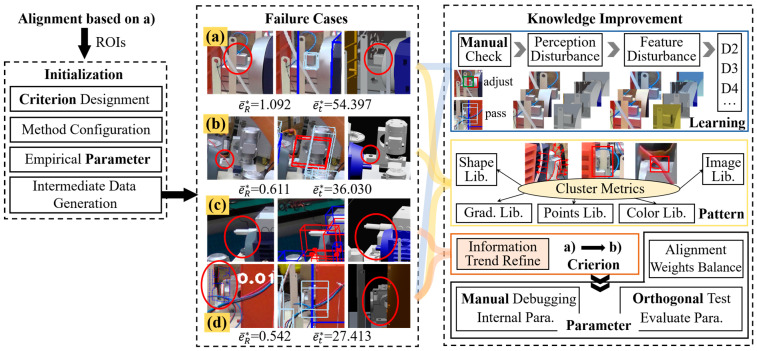
Inspection initialization, failure cases, and knowledge improvement: (**a**) mismatch caused by alignment deviation; (**b**) The difference between the reference pattern and the actual product (Red cable); (**c**) mismatch caused by local negligence in. alignment; and (**d**) errors may be overwhelmed by other objects during alignment, but it will be exposed in the inspection.

**Stage-B.** Inspection initialization and knowledge improvement based on the data generated by **Stage-A** are shown in Figure 12. The failure cases summarize the detection failure caused by alignment deviation, differences between virtual and reality, and local matching errors.

It can be observed that in practical industrial vision applications, a series of unpredictable situations may be implied in seemingly good results (e.g., $0.542^{{}^{\circ}}$, 27.413 mm in Figure 12), which further confirms the necessity of emphasizing interpretability, traceability, and improvability. Therefore, knowledge improvement based on **TESA** was performed as shown in Figure 12. For manual check step, only those cases with significant deviations will be manually corrected. In the pattern part, each sub-pattern space typically has its own dedicated measurement strategy, such as shape residual, cross entropy, Euclidean distance, etc. According to the field knowledge summarized in Section 3.3.2, **Criterion** was updated from 10-(a) to 10-(b), as shown in Figure 10. Additionally, an alignment balance strategy for improving case c was provided here. Specifically, this strategy can be achieved by increasing the optimization weights of objects located at the far end or around or reducing the geometric feature filtering work of high occluded objects. After the aforementioned work, the experimental results of stage-B were summarized in Table 5, where ${\bar{r}}_{n}^{PT}$ consists of the true positive rate ${\bar{r}}_{nr}^{PT}$ and false positive rate ${\bar{r}}_{nf}^{PT}$ (Recall rate).

The performance improvement in PSC is foreseeable, while the improvement in PT (from 0.712 to 0.845) proves that knowledge improvement has a positive effect on the practical application stage. However, according to our observations, pattern expansion and parameter adjustment on PSC increase the detection tolerance for pattern mismatch, resulting in ${\bar{r}}_{n}^{PSC}$ increasing from 0.778 to 0.894 while ${\bar{r}}_{nf}^{PT}$ also increased from 0.571 to 0.597. This phenomenon is actually due to overfitting in a specific pattern space when the observed information is limited. To address this issue, based on this study, it can be improved by accumulating experience in the target pattern space and adjusting the **Criterion** to increase the dimension of information perception.

Next, in the transitional **Stage-C,** while collecting experience with new patterns, we review the feedback of this system engineering on various types of anomalies. Simultaneously, selected [49] as the control group DL1, updated the templates of [50] as DL2, and arranged the state-of-the-art object recognizer Yolo7 [52] in DL3. To demonstrate the upper limit of the DL-based approaches performance, the dataset adopted here was generated from the pose truth of Stage-B (virtual-real ROIs and object bounding rectangle). additionally, the determination of whether it is an anomaly is based on the average confidence given on the test dataset.

**Figure 13:**
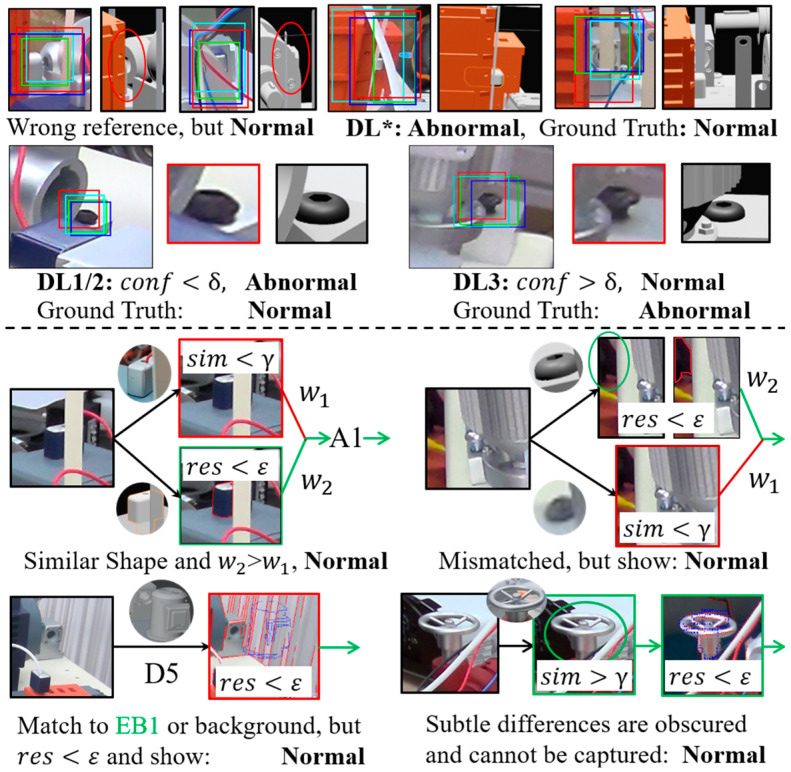
Failure cases of first contact with abnormal patterns. In the DL part, rectangular boxes of different colors represent the recognition results of different groups, and the corresponding relationships are detailed in Table 6. * abnormal.

Unexpectedly, compared to the results reported in ${\bar{r}}_{n}^{*}$ in Table 6, the performance of high-quality label-supervised learning models does not seem to have an absolute advantage over the previously encountered patterns. In summary, learning with reference patterns demonstrates superior discrimination ability for low-granularity position-sensitive anomalies (${\bar{r}}_{i}^{*}$ on ED), while mature recognition baselines demonstrate better potential performance for semantic-sensitive anomalies with high information volume (${\bar{r}}_{m}^{*}$ on EA/B). Despite the effectiveness being verified, Figure 13 demonstrates that there still exists a significant number of logical errors and unexpected errors hidden within the actual process data. More seriously, the analysis of the causes of these errors and the construction of avoidance methods are quite difficult. However, in the framework of this study, as shown in the second part of Figure 13, we can easily trace back these errors.

Similar to the previous stage, knowledge improvement work was carried out based on the TESA strategy. Abnormal cases were employed to construct hard samples and retrain D2, as well as to expand the pattern library. The **Criterion** was updated from (b) to (c) to increase the dimension of information observation to avoid misjudgment caused by incorrect matching in Figure 13, especially by incorporating D2 into alignment and inspection to alleviate the pressure of the evaluation parameter fitting. Finally, parameter debugging and orthogonal experiments on the improved framework were conducted. Correspondingly, the dataset of DL1 and DL2 were supplemented with OT and abnormal states were given greater weight during training.

After careful modifications, these methods have made significant progress compared to before, as shown in Table 7. In our framework, due to the control of **Criterion** and **Parameter** constraints, the results obtained at each stage are observable and interpretable, and some abnormal results given by D* module can be well-controlled. For DL-based approaches, the entire inspection of a complex assembled product heavily relies on positioning accuracy and dataset quality. By comparing the ${\bar{r}}_{i}^{*}$ in the table, system engineering based on industry experience still holds an advantage in identifying abnormal patterns related to highly specialized knowledge in industrial vision. The provided **Criterion** and methods may not be the optimal solution and can be further improved through PT or future accumulation. The reasons and basis for the revision of the **Criterion** is summarized in Table 8.

#### 4.2.3. Validation on Mobile Robot Capture System

**Figure 14:**
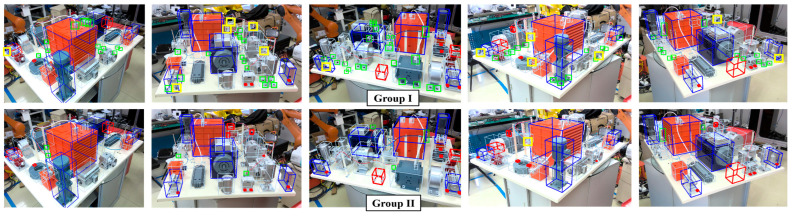
Localization and detection performance of mobile vision robot system with or without alignment. The dark blue mark was used to describe pose deviation, undetected normal objects were marked with bright green, and red and bright yellow indicate recognized and unrecognized defects, respectively.

The system model is AUBO AMR300-E5, where the positioning of the mobile base relies on laser SLAM and the indoor map is pre-built. The pose ground truth of $D_{4}$ is converted into a recognizable control command for the system. The error sources of the system include: a joint calibration error, system error, map construction and positioning error, and motion control error. In this scenario, we have observed that the real-time positioning error and the motion error caused by the idle movement of the base are the primary sources of errors. Therefore, in Table 9 and Figure 14, the first view captured after each vehicle reaches the target point was selected as the representative example. Due to the difficulty in obtaining real-time status, IoU was used to describe the potential deviation of the current system.

Upon tracing $\left({\bar{IoU}}_{EC},{\bar{IoU}}_{ED}\right)$, it can be observed that SLAM possesses the ability to perform real-time correction. However, as the system continues to operate for an extended period, error accumulation is inevitable, leading to a tendency for deviations to amplify. The results of Group II demonstrate the compatibility and transferability of the proposed framework. Through the comparison between I and II, we can see that, although it is possible to simplify **Connection** based on multi-device combination practically, complete alignment and inspection are still indispensable.

### 4.3. Validation on AR Projection under Different Object

**Figure 15:**
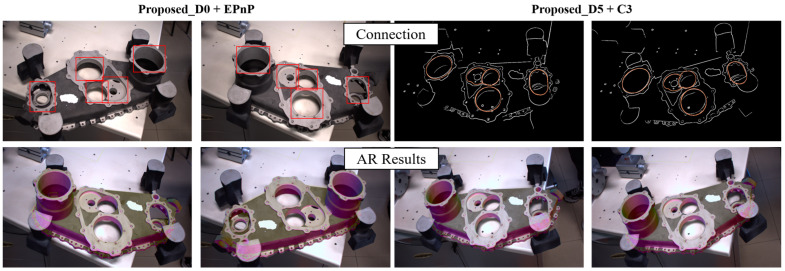
Examples of different Connection strategies and their final AR projection results.

The core problem of AR assisted assembly tasks is virtual–real alignment, which essentially still falls within the coverage scope of this framework. The suggested **Criterion** is shown in Figure 10a,b. D0 was retrained based on dataset $D_{5}$. EAx was selected as the significant element, and the five points associated with its recognition box were used as geometric features. The method pool remains unchanged, but **Parameter** needs to be debugged again on $D_{5}$. Through the trial operation on (a), it was found that the participation of EBx had a limited impact on overall accuracy, and therefore it was excluded in (b). Additionally, the pattern library (shape, gradient) of ECx was expanded in (b) to improve alignment performance. After the above work, two different **Connection** strategies were verified; the results are summarized in Table 10 and Figure 15.

Compared to Section 4.2, this case belongs to a common, simple case in industry. As can be seen in Figure 15, after alignment, the performance of the two connection strategies is similar and both demonstrate excellent projection effects. Considering that D5 can be replaced by many excellent feature extraction algorithms in the tracking field, the **Connection** with B4 + C3 may be a better choice from an efficiency perspective. To further improve accuracy, knowledge improvement work can be carried out from aspects such as incorporating the contour of EBx into the alignment process, expanding the pattern library of holes in various postures, etc.

### 4.4. Validation on Different Scenario and Task

In **Criterion-(a)**, a manual proxy based on multiple templates was constructed to preliminarily create a dataset. In **Criterion-(b)**, ES0/EA0/EBx were selected as the significant elements. For D0, the embedded constraints include (1) auxiliary tasks for recognizing ES0/EA0; (2) that the ordinates of the centroids of each element are close; and (3) that they are approximately parallel. These explicit constraints enable us to quickly train a specialized model without the risk of transferring large baseline models that are not applicable. The product model can be determined by the distribution of estimated key points and the corresponding 3D structure is loaded to perform **Connection** and **Alignment**. When the pattern library is insufficient, to prevent missed identification in perceived ROI, field knowledge of pin-tip reflection characteristics was leveraged to design a more reliable but inefficient method such as D5+A2+A1. After the expansion of the C3 and A2 pattern libraries, as well as the retraining of D0, the entire process was improved and simplified in **Criterion-(c)**. Considering the local positional uncertainty of the skewed pin, an empirical sampling+D1 **Criterion** was assembled based on (5) in Section 3.3.2, where the template used in D1 was replaced with a normal pin tip image.

The comparison between the method constructed according to this framework and advanced inspection schemes MTCI1 [80] and MTCI2 [22] for multi-type electrical connector is summarized in Table 11. ${\bar{Io\mathrm{U\_1}}}_{EC}^{*}$ is employed to compare the performance of each group’s initial matching work (recognition and registration in MTCI, **Connection** in this study). ${\bar{Io\mathrm{U\_2}}}_{EC}^{*}$ is adopted to evaluate the performance of the strategies implemented by each group (expand ROI and rematch in MTCI, **Alignment** + **Detection** in this study) to prevent missing recognition. Indicator ${\bar{Io\mathrm{U\_e}}}_{EC}^{*}$ refers to the IoU between the detected abnormal pin and the local search region.

**Figure 16:**
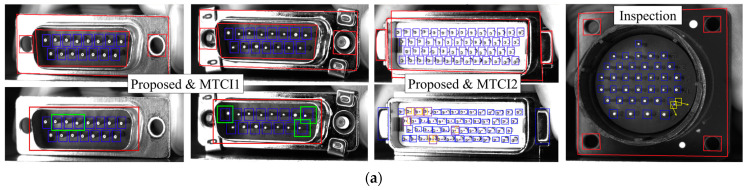

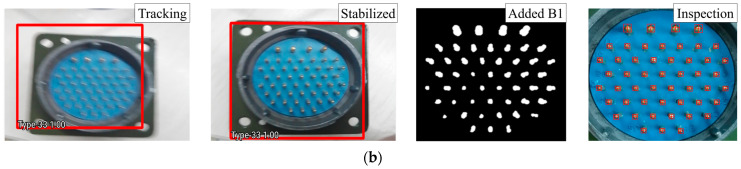
Validation on electronic product: (**a**) comparison of pin extraction performance and skewed pin inspection strategy, where the yellow box indicates the feasible sampling region for the skewed pin; and (**b**) tracking and inspection effects after migration to portable imaging, where the red box represents the recognition results.

The first row in Figure 16 shows the inapplicability of 2D-matching modes under large rotation angles. The method of combining registration and directly retraining the large baseline model, as utilized in MTCI1 and 2, does not fully account for task-related prior knowledge. This limitation results in a significant need for efforts directed towards improving and correcting outliers and missed recognitions, particularly when the target is imaged with a large rotation angle. Conversely, the proposed framework enables an easier and faster construction of a robust inspection scheme, leading to more accurate perceptual results. With the above foundation, we can generate a lightweight model suitable for real-time recognition from D0 through knowledge distillation. The image can be converted to grayscale and directly applied above works after **Parameter** debugging. Additionally, D5 and D1 can be replaced with B1, making it easier to determine the status of the pins from the color space. The effect of tracking and recognition is shown in the second row of Figure 16.

### 4.5. Knowledge Integration and Reapplication

Through the aforementioned works, pattern libraries, **Criterion,** and **Parameter** associated with task-objects, and the intermediate process data of different steps can be accumulated for different capture approaches and different scenarios. **Criterion** combinations that have been successfully validated on previous objects can be stored as new sub-patterns in the method pool. The original dataset, disturbance strategies for each stage, and the perturbed dataset are prepared to provide a foundation for more advanced DL models. The validated **Parameter** will be employed as additional expert knowledge to support similar tasks.

Overall, when migrating to new scenarios or tasks, the practical process can be summarized as follows:

1. Perform **Criterion** design (or directly call based on experience, or automatically recommend based on established pattern space (e.g., knowledge graph));
2. Build pattern connection and alignment;
3. Conduct internal debugging of **Criterion** + **Parameter**;
4. Implement joint testing of **Connection** + **Alignment**;
5. Determine if repetition is necessary.

With the continuous accumulation of experience, the above work will be progressively simplified and even directly applied in future encounters with new tasks.

## 5. Conclusions

We believe that liberating people from the complex physical and mental work to play a role in more critical positions, such as innovation and high-level decision-making, is one of the most important propositions towards next-generation intelligent vision and sensor technology. This paper focuses on the adaptability challenges brought by the transformation of manufacturing modes to traditional AVI, and proposes a common, knowledge-driven, generic vision inspection framework that includes three stages to standardize the inspection process, enhance the detection ability of composite vision task, and promote knowledge accumulation and reapplication within the industry. In the first stage, we demonstrated how to utilize common sense, such as the rigid structure stability of industrial products to construct constraints and seamlessly integrate them into both learning and non-learning processes for pattern connection. In the second stage, a multi-granularity, multi-pattern, hierarchical iterative alignment mechanism driven by structured tasks is designed to facilitate traceable and interpretable extraction of task-related objects. In the third stage, general detection items are normalized to the information mapping and processing of different sub-pattern spaces or their combinations, and TESA knowledge improvement and accumulation strategy is designed to ensure internal improvability and transferability between applications.

For a composite appearance inspection task on an assembled product consisting of 113 objects, the inspection pipeline generated based on the proposed framework demonstrates the advantages of traceability, improvability, and weakly annotated dependency compared to advanced deep learning methods. Moreover, compared to the state-of-the-art recognition model with truth labels, the better potential and performance demonstrated in this study on the positioning accuracy and position-sensitive anomaly detection indicates its effectiveness. In addition, the validation of other capture approaches, tasks, and objects preliminarily verifies the adaptability of this study, and the significantly reduced deployment efforts prove the importance and necessity of knowledge accumulation.

It should be noted that this study is only applicable to situations where common knowledge is consistent, including where reference patterns can be provided (3D models, drawings), rigid products, non-precision measurements, etc. In terms of practical application, it is particularly suitable for the customized production process of single piece and small batch in the aerospace field. On the other hand, it is undeniable that the adjustment of **Criterion** and **Parameter** in knowledge improvement still rely on humans, and that, perhaps, the workload of improvement is greater than that of data annotation. Therefore, in future work, we plan to conduct validation in more practical scenarios to enrich our experience in **Criterion** design and encapsulate validated **Parameter**, sub-patterns, and intermediate data. Furthermore, we will construct a knowledge graph for AVI, allowing us to establish a recommendation system to assist operators in improving designment and decision-making when dealing with new tasks.

## Figures and Tables

**Table 1:** The number of times an abnormal object can be recognized by the naked eye in the dataset.

ID	EB2	EA6	ED49	ED50	ED13	ED14	EC11	ED43	ED44	EB5	ED25	ED26
$D_{2}$	58	97	73	45	41	35	88	43	69	77	52	53
$D_{3}$	41	72	36	53	31	30	61	35	48	61	41	39

**Table 2:** Framework configuration.

Item	Configurations
Significant elements	ES0, ES1
Feature design	Projected cuboid described by 8 + 1 2D points
Constraints	Auxiliary recognition task Approximate parallel constraint
Post processing	Weight adjustment [82]
A1	CPD [88]
A2	Edge-based [96]
A3	Weighted-ICP [81]
A4	Shape fitting algorithm set
B1	Color segmentation, K-means
B2	SLIC [97]
B4	HOG, Canny, Sobel
C3	[98] with multiple templates
C4	SURF [99] with parallel constraints

**Table 3:** Deployment and execution of detection experiments.

Stage	Description	Datasets	Criterion	Indicators
A	Connection and alignment	PSC	10-(a)	${\bar{e}}_{R}^{*}$, ${\bar{e}}_{t}^{*}$, ${\bar{IoU}}_{EC}^{*}$, ${\bar{IoU}}_{ED}^{*}$, ∗=PSC,PT
B	Inspection initialization Knowledge improvement Connection and alignment	PSC	10-(b)	${\bar{e}}_{R}^{*}$, ${\bar{e}}_{t}^{*}$, ${\bar{IoU}}_{EC}^{*}$, ${\bar{IoU}}_{ED}^{*}$, and ${\bar{r}}_{n}^{*}$, ∗=PSC,PT
C	Connection, alignment and inspection	TO	10-(b)	${\bar{IoU}}_{EC}^{*}$, ${\bar{IoU}}_{ED}^{*}$,∗=TO, ${\bar{r}}^{*}$, ∗= TO, PT
D	Knowledge improvement Connection, alignment and inspection	PSC, TO	10-(c)	all ${\bar{r}}^{*}$, ∗= ALL

**Table 4:** Performance of Stage-A of the experiment.

	Connection	Alignment	Group I	Group II	Group III	Group IV	Group V
${\bar{e}}_{R}^{PSC}$(°)	2.678	0.817	3.357	1.594	1.613	2.181	3.895
${\bar{e}}_{t}^{PSC}$(mm)	142.196	35.304	163.623	64.725	62.014	97.864	165.074
${\bar{IoU}}_{EC}^{PSC}$	0.553	0.805	0.515	0.700	0.688	0.612	0.394
${\bar{IoU}}_{ED}^{PSC}$	0.374	0.643	0.336	0.469	0.460	0.405	0.177
${\bar{e}}_{R}^{PT}$(°)	2.771	1.342	3.429	1.767	1.838	2.475	4.023
${\bar{e}}_{t}^{PT}$(mm)	153.584	59.641	169.240	69.802	74.125	114.523	181.451
${\bar{IoU}}_{EC}^{PT}$	0.547	0.721	0.497	0.673	0.662	0.577	0.367
${\bar{IoU}}_{ED}^{PT}$	0.369	0.489	0.314	0.432	0.419	0.398	0.113

**Table 5:** Performance of Stage-B of the experiment.

	Before		After Improvement					
PSC	${\bar{r}}_{n}^{PSC}$		${\bar{e}}_{R}^{PSC}$	${\bar{e}}_{t}^{PSC}$	${\bar{IoU}}_{EC}^{PSC}$	${\bar{IoU}}_{ED}^{PSC}$	${\bar{r}}_{n}^{PSC}$	
	0.778		0.517	27.304	0.882	0.730	0.894	
PT	${\bar{r}}_{nr}^{PT}$	${\bar{r}}_{nf}^{PT}$	${\bar{e}}_{R}^{PT}$	${\bar{e}}_{t}^{PT}$	${\bar{IoU}}_{EC}^{PT}$	${\bar{IoU}}_{ED}^{PT}$	${\bar{r}}_{nr}^{PT}$	${\bar{r}}_{nf}^{PT}$
	0.712	0.571	1.034	52.308	0.755	0.492	0.845	0.597

**Table 6:** State recognition performance of Stage-C of the experiment.

	${\bar{r}}_{n}^{TO}$	${\bar{r}}_{m}^{TO}$		${\bar{r}}_{w}^{TO}$	${\bar{r}}_{i}^{TO}$		${\bar{r}}_{n}^{PT}$	${\bar{r}}_{m}^{PT}$		${\bar{r}}_{w}^{PT}$	${\bar{r}}_{i}^{PT}$	
		EA/B	ED		EB	ED		EA/B	ED		EB	ED
Proposed (Green)	0.856	0.671	0.736	0.585	0.104	0.244	0.845	0.425	0.642	0.538	0.049	0.270
DL1 (Red)	0.797	0.665	0.704	0.724	0.247	0.387	0.766	0.681	0.692	0.670	0.213	0.325
DL2 (Blue)	0.812	0.697	0.821	0.805	0.260	0.401	0.773	0.664	0.808	0.714	0.213	0.399
Yolo7 (Cyan)	0.914	0.742	0.805	0.862	0.026	0.069	0.901	0.735	0.783	0.835	0.016	0.074

**Table 7:** State recognition performance of Stage-D of the experiment.

	${\bar{r}}_{n}^{TO+PSC}$	${\bar{r}}_{m}^{TO}$	${\bar{r}}_{w}^{TO}$	${\bar{r}}_{i}^{TO}$	${\bar{r}}_{n}^{PT}$	${\bar{r}}_{m}^{PT}$	${\bar{r}}_{w}^{PT}$	${\bar{r}}_{i}^{PT}$
Proposed+	0.981	0.984	0.967	0.908	0.932	0.936	0.956	0.884
DL2+	0.917	0.930	0.927	0.786	0.825	0.910	0.945	0.741
Yolo7+	0.943	0.962	0.959	0.721	0.938	0.944	0.967	0.683

**Table 8:** Reasons for modifying Criterion.

Stage	Criterion Scheme	Object ID	Expert Experience
A	At first, there were only a few samples, and the framework built through experience and expertise served as the agent for data acquisition.		
	(a)-Alignment	ES0/ES1/EBx/EAx	Extracting domain independent geometric information in the case of a small number of samples and no annotation.
		EA3/EA18/EA16	As the significant feature, color-region can reduce the amount of information received by A2.
		ECx	The small silver-gray plug has the characteristics of low information volume and consistent texture. Simple shape allows us to pre-built a shape library.
		EDx	The materials of standard parts are uniform. The small size of the fastener makes its features clustered and pattern single.
B, C	After knowledge improvement, the state recognition pipeline is built according to the collected samples, and the general task model and various pattern libraries are enabled.		
	(b)-Alignment	ES0/ES1/EBx	A1 can be maintained under the constraint of Connection.
		ECx	It is found that B2 is easy to gather the plug and cable (bracket) together, so B4 is adopted to extract the gradient, and A2’s shape library is enriched.
		EDx	A4 is added to prevent the shadow part from being miscalculated as part of the shape, resulting in centroid offset.
	(b)-Detection	EA0/EA1/…/EA14	D5 is used to mitigate noise. Another purpose of A2 besides matching is to enrich the shape Library.
		EA5/EB3	The texture is complex but regular, which makes the geometric matching prone to bias, but the analysis in gradient mode will benefit from it.
		EA8/EA12/EB1/EB2	Significant geometric features (box structure).
		EA3/EA16/EA18	Significant visual features (Color)
		EA10/EA15/EA17	Significant geometric features. (Quasi circular structure)
		EB4/EB5	The pattern change under visible conditions is single, so C3 is configured.
		ECx	C3 is added to prevent some missed detections.
		EDx	A2 replaces A4 used in alignment to determine the position of object, and C3 is assembled to assist in determining whether the object exists.
D	(c)-Alignment	EBx	By observing Alignment of (b), it is found that the defect of EBx will cause large matching deviations, so C4 is configured to limit the search of A1.
		EAx/ECx	D2 is introduced to provide observation of semantic space to prevent local matching errors.
	(d)-Detection	EA0/EA1/…/EA14	Configure D3 to enrich observation space.
		EA5/EB3	Configure A1 to add position sensitivity.
		EA12	Due to the special shape and invisibility (based on experience), recognition can be completed only by C3.
		EB4/EB5	Although significant shapes are easy to fit, in practice, it is quite easy to make matching errors under the influence of cables and brackets, so D2 and D5 are added.
		EB1/EB2	Configure D3 to enrich observation space.

**Table 9:** Performance on a planning-based mobile vision robot system.

		$D_{4}^{1}$	$D_{4}^{2}$	$D_{4}^{3}$	$D_{4}^{4}$	$D_{4}^{5}$
Potential deviation $\left({\bar{IoU}}_{EC},{\bar{IoU}}_{ED}\right)$		(0.623, 0.403)	(0.636, 0.407)	(0.579, 0.364)	(0.659, 0.411)	(0.655, 0.412)
Visible Number	Normal	54	60	61	50	49
	Defects	4	8	7	7	6
Group I	Normal	42	44	35	37	36
	Defects	3	3	6	3	5
Group II	Alignment	(0.907, 0.812)	(0.918, 0.808)	(0.911, 0.815)	(0.933, 0.820)	(0.929, 0.812)
	Normal	52	58	58	50	47
	Defects	4	8	7	6	6

**Table 10:** Validation on actual AR-assisted assembly cases.

	${\bar{e}}_{R}^{D_{5}}({}^{\circ})$	${\bar{e}}_{t}^{D_{5}}(mm)$	${\bar{IoU}}_{EB}^{D_{5}}$	${\bar{IoU}}_{EC}^{D_{5}}$
Proposed_(a) D5 + C3	0.986	43.423	0.874	0.561
Proposed_(a) D0 + EPnP	1.021	52.786	0.833	0.515
Proposed_(b)	0.753	37.665	0.927	0.722

**Table 11:** Performance comparison on the pin-tip extraction of multi-type electrical connectors.

	${\bar{Io\mathrm{U\_1}}}_{EC}^{D_{6}}$	${\bar{Io\mathrm{U\_2}}}_{EC}^{D_{6}}$	${\bar{Io\mathrm{U\_1}}}_{EC}^{D_{7}}$	${\bar{Io\mathrm{U\_2}}}_{EC}^{D_{7}}$	${\bar{Io\mathrm{U\_e}}}_{EC}^{D_{7}}$
MTCI	0.625	0.854	0.691	0.837	0.219
MTCI2	0.723	0.878	0.648	0.844	0.286
Proposed	0.705	0.963	0.722	0.955	0.425

## Data Availability

The original contributions presented in the study are included in the article, further inquiries can be directed to the corresponding author.
